# Supplementary material for: Complexome profiling of the Chlamydomonas psb28 mutant reveals TEF5 as an early PSII assembly factor
Source: Plant Cell. 2025 Mar 17;37(6):koaf055. doi: 10.1093/plcell/koaf055 (PMC12164590; doi:10.1093/plcell/koaf055)
Supplement: koaf055_Supplementary_Data [file koaf055_supplementary_data.zip › psb28_interactive.zip!/plots/intensity/qualityPlots/peptides/MIP1_Cre12.g549300.t1.2.html]

### Cre12.g549300.t1.2

Trivial name: MIP1  
SeqCoverage [median]: 24.30  
Mass: 31.53 kDa  
  
 

| Sequence | MissedCleavage | Mass [Da] | wtIntensity | muIntensity | Score |
| --- | --- | --- | --- | --- | --- |
| GYQDEGQGLVELGTAHDAVPK | 0 | 2183.05 | 469.72 | 16736.28 | 96.79 |
| LRPYLDPSGQVK | 1 | 1371.75 | 2816.37 | 10910.52 | 79.82 |
| SLEVQSK | 0 | 789.42 | 1629.40 | 4895.37 | 111.01 |
| SLEVQSKYDR | 1 | 1223.61 | 419.64 | 0.00 | 47.82 |
| TTAQPKYGDDDDLLPVVLER | 1 | 2244.13 | 0.00 | 2165.63 | 57.41 |
| YDRGYQDEGQGLVELGTAHDAVPK | 1 | 2617.24 | 0.00 | 6951.45 | 66.30 |
| YGDDDDLLPVVLER | 0 | 1617.79 | 601.99 | 13718.21 | 218.01 |
| YLEKAGVMQK | 1 | 1165.62 | 0.00 | 689.55 | 76.83 |
|  |  |  | 5937.1 | 56067.0 |  |
